# Roles of bacterial extracellular vesicles in systemic diseases

**DOI:** 10.3389/fmicb.2023.1258860

**Published:** 2023-09-28

**Authors:** Yanzhen Wang, Xinghong Luo, Xiaozhen Xiang, Chunbo Hao, Dandan Ma

**Affiliations:** ^1^Department of Endodontics, Stomatological Hospital, School of Stomatology, Southern Medical University, Guangzhou, Guangdong, China; ^2^Department of Stomatology, Nanfang Hospital, Southern Medical University, Guangzhou, Guangdong, China; ^3^Hainan General Hospital (Hainan Affiliated Hospital of Hainan Medical University), Haikou, Hainan, China

**Keywords:** bacterial extracellular vesicles, outer membrane vesicles, membrane vesicles, interaction, pathogenesis, systemic diseases

## Abstract

Accumulating evidence suggests that in various systems, not all bidirectional microbiota–host interactions involve direct cell contact. Bacterial extracellular vesicles (BEVs) may be key participants in this interkingdom crosstalk. BEVs mediate microbiota functions by delivering effector molecules that modulate host signaling pathways, thereby facilitating host–microbe interactions. BEV production during infections by both pathogens and probiotics has been observed in various host tissues. Therefore, these vesicles released by microbiota may have the ability to drive or inhibit disease pathogenesis in different systems within the host. Here, we review the current knowledge of BEVs and particularly emphasize their interactions with the host and the pathogenesis of systemic diseases.

## 1. Introduction

Bacterial extracellular vesicles (BEVs) are nanosized lipid vesicles with a particle size of 20–250 nm that are secreted by bacteria during growth. In 1963, the presence of BEVs was first observed in the cell wall of gram-negative bacteria by electron microscopy, and initial research primarily focused on BEV functions. Initially, they were viewed as cellular debris that occurred after dead cells degraded (Bishop and Work, 1965). Nonetheless, due to cargo analysis of BEVs and the discovery of their biogenesis mechanism, they are now regarded as contributors to physiological and pathological processes that lead to the occurrence and development of systemic diseases.

Although gram-negative and gram-positive bacteria have different vesicle secretory pathways due to different cell wall structures, as vectors of diverse bioactive compounds, BEVs participate in bacterial intraspecific and interspecific communication and interactions with hosts, including horizontal gene transfer, the killing of competing bacteria, phage neutralization and the delivery of virulence factors to host cells (Tzipilevich et al., 2017; Wei et al., 2022). In addition, components such as lipopolysaccharides (LPS) and peptidoglycan carried by vesicles are naturally immunogenic and can be recognized by host cell pathogen-recognition receptors (PRRs) to activate signaling pathways, induce cytokine production, and play physiological and pathological roles similar to those of parent bacteria (Dauros-Singorenko et al., 2020). Emerging studies have revealed that BEVs are involved in diseases of various systems of the human body, promoting bacterial infections and pro−/anti-inflammatory responses to drive the onset and progression of systemic diseases, such as autoimmune diseases, inflammatory bowel disease (IBD), liver diseases, allergic diseases, and metabolic syndromes such as diabetes.

In this review, we discuss the key functions of BEVs. Furthermore, we summarize the pathways of BEV synthesis and the mechanisms through which BEVs released by commensal and pathogenic bacteria are recognized by host PRRs to initiate inflammatory responses or mediate immunomodulation. Moreover, we highlight their biological role in microbiota-host interactions, in addition to their role in the pathogenesis of diseases of specific systems, namely, the nervous, digestive, circulatory, respiratory and motor systems.

## 2. Contents and biogenesis of bacterial extracellular vesicles

**Figure 1:**
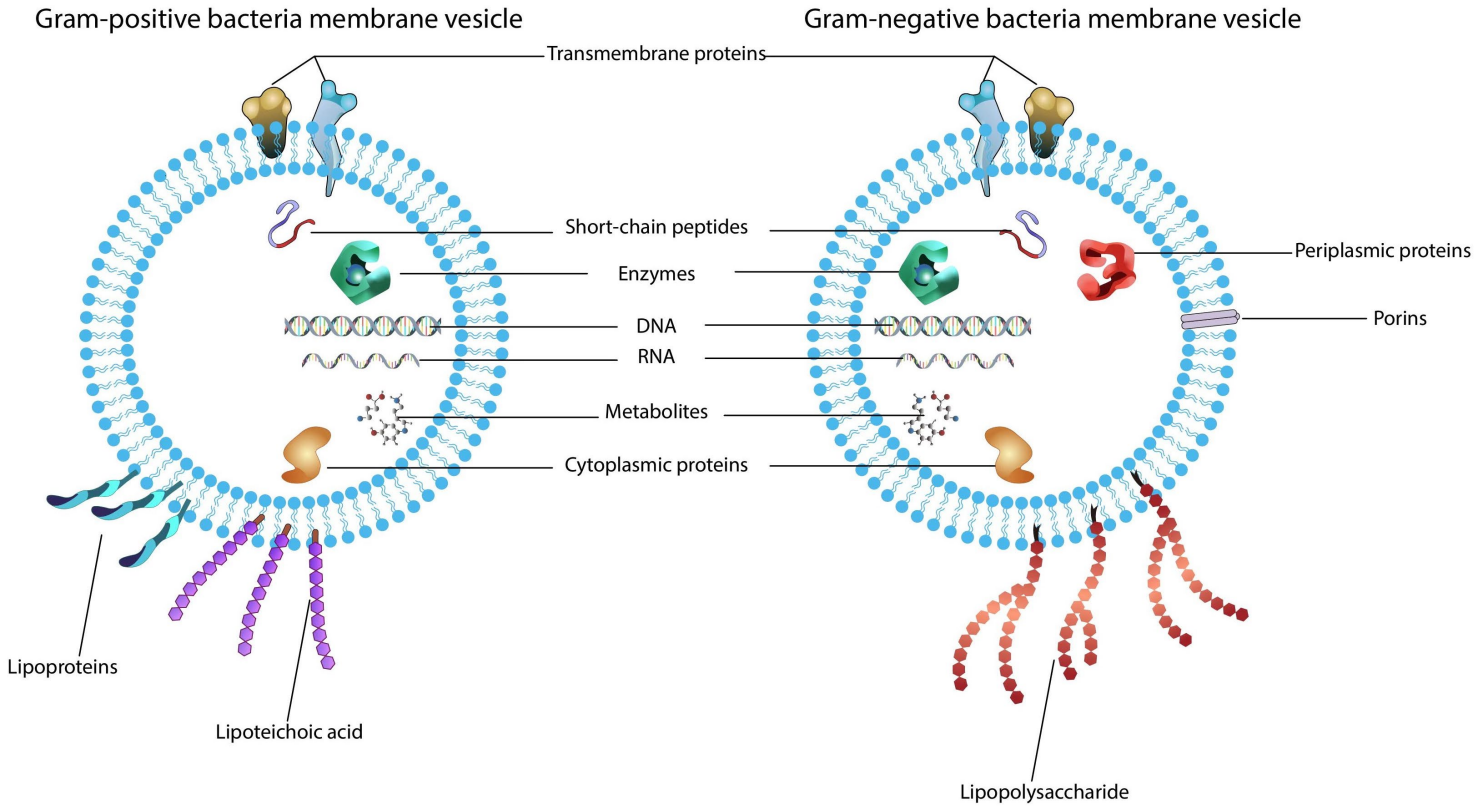
Structure and content of bacterial extracellular vesicles. (1) Gram-positive bacteria produce vesicles that carry bacterial cargo including transmembrane proteins, enzymes, toxins, peptidoglycan, lipoproteins, lipoteichoic acids and nucleic acids. (2) Outer membrane vesicles secreted by gram-negative bacteria also contain cargo composed of the parental bacterial components within a lipid membrane, such as proteins, lipids, peptidoglycan, and nucleic acids.

The structures of gram-positive bacteria and gram-negative bacteria have obvious differences, as do their released vesicles (Figure 1). Currently, there are different opinions on the mechanism of BEV biosynthesis; however, a consistent conclusion is that the formation and release of BEVs is not a random act but an ordered regulatory process (Schwechheimer and Kuehn, 2015).

### 2.1. Contents and biogenesis of gram-negative bacterial outer membrane vesicles

The cell wall of gram-negative bacteria consists of an outer membrane, an inner membrane, and a peptidoglycan layer between them. Gram-negative bacteria release the outer membrane and cytoplasmic contents through the formation of a nanosized spherical structure with diameters in the range of 10–300 nm, whose composition is analogous to the outer membrane, so they are called outer membrane vesicles (OMVs). OMVs basically consist of proteins, virulence factors, lipids, and peptidoglycan (Santos et al., 2018; Toyofuku et al., 2019). Most of the proteins in OMVs are virulence-related proteins, and the lipids of OMVs are primarily LPS and phospholipids (Liu et al., 2019).

Gram-negative bacteria produce OMVs mainly in two ways: (1) through an imbalance in biosynthetic peptidoglycan or when hydrophobic molecules are embedded in the outer membrane, thereby causing cell membrane destabilization, which ultimately leads to the vesiculation of the outer membrane: the reduction of crosslink between peptidoglycan and the outer membrane induces the expansion of the outer membrane; the accumulation of bacterial cell wall peptidoglycan fragments increases peripheral pressure so that the outer membrane is bent to generate OMVs; and the accumulation of molecules that increase membrane curvature such as LPS can trigger membrane swelling (Arunmanee et al., 2016; Avila-Calderón et al., 2021); and (2) the generation of explosive outer membrane vesicles (EOMVs) and outer-inner membrane vesicles (OIMVs) can occur *via* cell lysis (Toyofuku et al., 2019). In recent years, a general mechanism has emerged that can explain how OMVs form in different environments, and the destruction of the highly conserved VacJ/Yrb ABC transport system may be a core mechanism involved in this process (Roier et al., 2016). These mechanisms are based on the common hypothesis that vesicles bulge from the outer membrane, and the destruction of the crosslink between peptidoglycan and the outer membrane or the increased extrusion pressure causes the outer membrane to separate from the peptidoglycan layer and release in the form of OMVs (Toyofuku et al., 2017).

### 2.2. Contents and biogenesis of gram-positive bacterial membrane vesicles

Gram-positive bacteria are a class of bacteria surrounded by a plasma membrane and a thick peptidoglycan layer. Due to the lack of an outer membrane and a thick cell wall, they were initially thought to be unable to produce and secrete EVs. In 2007, researchers isolated vesicles from mycobacteria and conducted extensive proteomic studies of their components, confirming that gram-positive bacteria also release EVs (Lee et al., 2009). Researchers then found that the vesicles of gram-positive bacteria are produced from the inner membrane and secreted through the peptidoglycan layer to the surrounding environment; therefore, these vesicles are usually called cytoplasmic membrane vesicles (CMVs) or membrane vesicles (MVs) (Brown et al., 2015). MVs contain cell membrane and cytoplasmic components, and periplasmic components are unique to MVs and not to OMVs (Toyofuku et al., 2017). MVs are approximately 20 ~ 400 nm in diameter and consist of membrane-associated proteins, cytoplasmic proteins, peptidoglycan, and lipoteichoic acid (Brown et al., 2015; Kopparapu et al., 2021).

The pathway by which gram-positive bacteria form MVs is primarily through endolysin-triggered death lysis, which is called bubbling cell death (Toyofuku et al., 2019). Studies have revealed that endolysin expressed by pro bacteriophages creates pores in the peptidoglycan layer of the cell wall; therefore, the material in the cytoplasm bulges outward and is released in the form of MVs; meanwhile, a few of them are secreted *via* encapsulation from the remaining peptidoglycan layer (Toyofuku et al., 2017; Jeong et al., 2022). Explosive cell lysis is another form of MV biogenesis (Jeong et al., 2022). Regarding the special type of EVs found in recent years, namely, the tubular membrane structure, local lysis of the cell wall may lead to blistering of the cytoplasmic membrane, which forms a nanotube structure as a bridge for material exchange between bacteria (Baidya et al., 2018). Although there is currently a lack of definitive evidence regarding how gram-positive bacteria bypass the thick cell wall to produce MVs, some underlying mechanisms can be investigated based on studies of vesicle composition, morphology, etc. (Kalra et al., 2016).

### 2.3. Conclusions on BEV biogenesis

Although the generation of BEVs is an energy-consuming process, this secretory mode has irreplaceable advantages in protecting cargo from degradation by extracellular proteases and triggering receptor-mediated signal transcriptional induction in host cells (Kaparakis-Liaskos and Ferrero, 2015). The biogenesis and composition of BEVs is dependent on the milieu to which the bacterium is exposed, and how vesicle formation and content shift in response to varying biological environments needs to be investigated to identify their specific functions.

## 3. The interaction between BEVs and host cells

**Figure 2:**
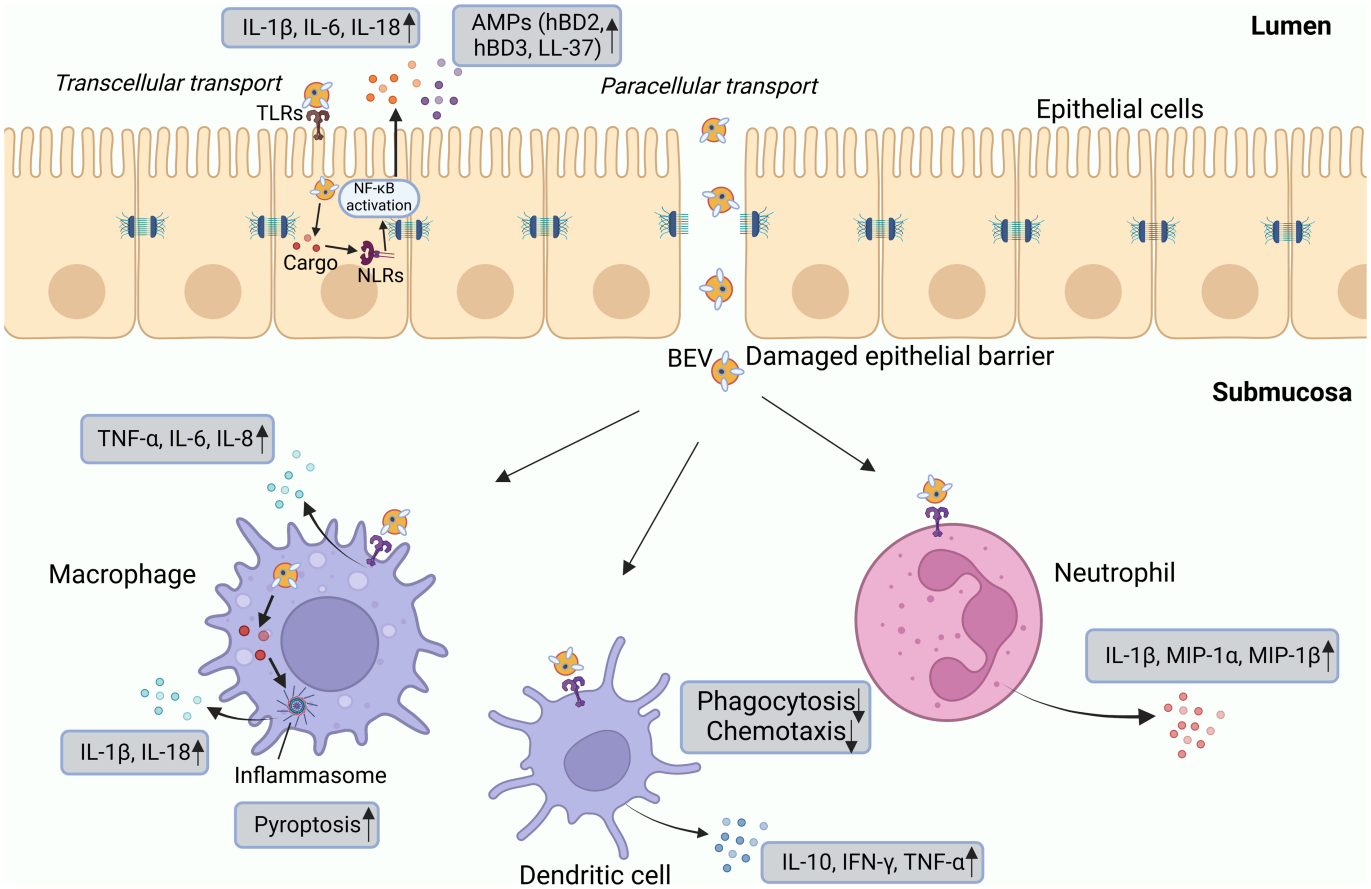
Interactions of BEVs with epithelial and innate immune cells. BEVs can directly interact with epithelial cells or PRRs to promote the production of IL-1β, IL-6, IL-8, and AMPs. Cargo delivered by BEVs is detected by intracellular NLRs and then activates signal transduction pathways. The interaction between BEVs and epithelial cells can damage tight junctions, facilitating the delivery of BEVs and the release of cargo components into the submucosa, where they can directly interact with immune cells. Macrophages produce inflammatory cytokines primarily in response to the activation of TLR2 and TLR4. Dendritic cells, upon stimulation by BEVs, enhance the release of IL-10, IFN-γ, and TNF-α. Neutrophils secrete proinflammatory cytokines such as IL-1β, MIP-1α, and MIP-1β. BEVs also promote pyroptosis and inhibit the chemotaxis and phagocytosis of immune cells. AMP, antimicrobial peptide; HBD, human β-defensin; MIP, macrophage inflammatory protein; IFN, interferon; IL, interleukin; LPS, lipopolysaccharide; NF-κB, nuclear factor-κB; TNF, tumor necrosis factor.

BEVs can be released at all stages during bacterial growth as a secretory system that influences the communication and interaction between hosts and bacteria. Pathogen-associated molecular pattern (PAMP) contents of BEVs enable them to bind to PRRs on the membrane surface and in the cytoplasm of immune cells and nonimmune cells, thereby activating downstream inflammatory signaling pathways (Alvarez et al., 2016; Table 1). After BEVs enter host cells, they can transmit immunogenic protein components, DNA, and sRNAs into recipient cells to prime the host immune responses (Bitto et al., 2021; Figure 2).

**Table 1:** Pathogenic PAMPs, host cell PRRs, and relevant signaling pathways.

PAMPs	Class of PRR	PRR location	Signaling pathway
Lipopolysaccharide (LPS)	TLR4, NLRP3	Cell membrane surface/cytoplasmic	NF-κB signaling, NLRP inflammasome, TRIF signaling
Outer membrane protein (Omp)	TLRs	Cell membrane surface	NF-κB signaling
Porin	TLRs	Cell membrane surface	NF-κB signaling
Lipoteichoic acid (LTA)	TLRs	Cell membrane surface	NF-κB signaling
Peptidoglycan	TLRs, NLRs NLRPs	Cell membrane surface/cytoplasmic	NF-κB signaling, NLRP inflammasome
Flagellin	TLR5, NLRC4	Cell membrane surface/cytoplasmic	NF-κB signaling
Nucleic acid	TLRs, NLRs, NLRPs, AIM2, STING	Cell membrane surface/cytoplasmic	NF-κB signaling, IRF3 signaling, NLRP inflammasome, AIM inflammasome, IRF3 signaling
Protein	TLRs, NLRs, NLRPs	Cell membrane surface/cytoplasmic	NF-κB signaling, MAPK signaling, IRF signaling, NLRP inflammasome

TLR, toll-like receptor; NLR, nucleotide-binding oligomerization domain-like receptor; NLRP, NLR thermal protein domain associated protein; AIM, absent in melanoma; STING, stimulator of interferon genes.

### 3.1. The entry of BEVs into mammalian host cells

#### 3.1.1. The entry modes of BEVs into host cells

The entry of gram-negative bacterial OMVs into host cells *via* multiple pathways has been demonstrated, allowing their cargo to be detected by PRRs, and subsequently activating a series of signaling pathways. In phagocytic cells, phagocytosis is the major route to internalize OMVs (O'Donoghue and Krachler, 2016). In nonphagocytic cells, other endocytic routes affect the entry of OMVs, namely, clathrin-, caveolin-and lipid raft-mediated endocytosis and macropinocytosis, direct membrane fusion, and receptor-mediated uptake (O'Donoghue and Krachler, 2016). The biophysical properties inherent to OMVs including the surface lipid phase and membrane curvature might enable them to enter or fuse with early endosomes and then disintegrate and release their contents into the cytoplasm (Mulcahy et al., 2014; Behrens et al., 2021). Instead, early endosomes can transform into late endosomes and fuse with lysosomes, causing degradation of BEVs (Mulcahy et al., 2014). Subsequently, BEV contents released into the cytosolic space can activate signaling pathways and induce pro−/anti-inflammatory responses (O'Donoghue and Krachler, 2016).

There are relatively few studies on the internalization pathways of MVs derived from gram-positive bacteria. Recent studies have found that MVs enter host cells mainly *via* clathrin-dependent endocytosis, dynamin-dependent endocytosis, and membrane fusion (Bajic et al., 2020; Wang et al., 2020).

#### 3.1.2. Factors that influence BEV uptake

Several factors seem to influence the mode and rate of BEV uptake, such as the size and composition of BEVs. For instance, smaller BEVs (20–100 nm) preferentially enter recipient cells *via* caveolin-mediated endocytosis, clathrin-mediated endocytosis can be utilized by BEVs with diameters ranging from 20 to 250 nm, and macropinocytosis appears to be effective for larger BEVs (90–450 nm). Clathrin-mediated endocytosis can be utilized by BEVs with diameters ranging from 20 to 250 nm (Weiner et al., 2016; Zhang et al., 2019). Toxins can serve as BEV adhesins and allow their internalization *via* ligand-receptor interactions. Additionally, BEVs could utilize complementary mechanisms to promote their entry. For example, the uptake of OMVs with O antigen is lipid raft-dependent, while OMVs lacking O antigen alternatively select clathrin-mediated endocytosis (O'Donoghue et al., 2017).

#### 3.1.3. BEV uptake conclusion

Bacterial extracellular vesicles enter host cells with multiple uptake mechanisms among vesicles from different species of bacteria and even among that from the same bacterium. The quantifiable and dynamical assay of uptake pathways will be important in the illustration of bioprocesses that underlie the bacteria-host interactions, but also in the design of BEV-engineered delivery vectors and improvement of their treatment efficiency based on their entry into target cells.

### 3.2. Inflammatory responses triggered by BEVs

#### 3.2.1. BEVs are sensed by PRRs on the cell membrane

PRRs present on the surface of immune cells can sense microbial-associated molecular patterns (MAMPs)/PAMPs carried by BEVs, activate signaling pathways, promote the release of proinflammatory cytokines, and trigger inflammatory responses (Cecil et al., 2017). For instance, Toll-like receptors (TLRs) of microglia and macrophages can recognize LPS, lipoproteins, flagellin and DNA carried by OMVs to release cytokines such as TNF-α and IL-10 (Balhuizen et al., 2022; Liu et al., 2022). BEVs can also be sensed by nonimmune cells. LPS carried by *Pseudomonas aeruginosa* OMVs triggers the immune response in epithelial cells through the MyD88-dependent TLR4 signaling pathway and promotes the expression of IL-8 in lung epithelial cells (Vitse and Devreese, 2020). DNA, RNA and peptidoglycan cargo in *Staphylococcus aureus* MVs activated several TLRs and nucleotide-binding oligomerization domain (NOD) 2 signaling and promoted cytokine and chemokine release by epithelial cells (Bitto et al., 2021). Upon stimulation by BEVs, cell surface PRRs also modulate antimicrobial peptide secretion, as evidenced by vesicles derived from *Helicobacter pylori*, *P. aeruginosa*, *Neisseria gonorrhoeae* and *C. jejuni* that induced the production of human β-defensins (hBD2, hBD3) and LL-37 by human gastric epithelial cells (Elmi et al., 2012). These *in vitro* studies revealed some of the mechanisms underlying bacteria-host interactions, whereas in the context of *in vivo* infections, we need to further clarify the mechanisms by which host cells detect BEVs to trigger immune responses.

#### 3.2.2. BEVs are sensed by PRRs in the cytoplasm

Although most studies have reported that BEVs activate PRRs on the cell surface, PAMPs carried by BEVs can also be perceived and recognized by PRRs in the cytoplasm of host cells, thereby activating intracellular innate immunity and promoting the assemble assembly of inflammasomes (Vanaja et al., 2016).

##### 3.2.2.1. Canonical inflammasome activation

Currently, four types of inflammasomes have been reported, namely, NLRP1, NLRP3, NLRC4, and AIM2, which eventually activate caspase-1 and induce the production of proinflammatory cytokines (Elizagaray et al., 2020; Johnston et al., 2021). The caspase-1-dependent process is called canonical inflammasome activation. Studies have shown that microbial DNA and flagellin carried by OMVs can activate inflammasome signaling in macrophages, as well as in *in vivo* models, inducing caspase-1-mediated pyroptosis and TNF-α, IL-1β, and IL-18 secretion (Elizagaray et al., 2020; Yang et al., 2020). Likewise, MVs produced by gram-positive bacteria delivering nucleic acids and peptidoglycan to epithelial cells can activate the NLRP3 inflammasome and caspase-1 and induce IL-1β and IL-18 production in macrophages (Wang et al., 2020).

##### 3.2.2.2. Noncanonical inflammasome activation

The activation of the noncanonical inflammasome depends on caspase-11 (mice) or caspase 4/5 (human) (Elizagaray et al., 2020). OMVs transport LPS into host cells and activate caspase-11 *via* guanylate-binding proteins (Santos et al., 2018). Active caspase-11 enhances gasdermin D pore formation in the cell membrane of macrophages, causing NLRP3 inflammasome-mediated pyroptosis (Kayagaki et al., 2015; Vanaja et al., 2016). In human monocytes, *P. aeruginosa* OMVs activated noncanonical inflammasomes in a caspase-5-dependent manner (Bitto et al., 2018).

#### 3.2.3. Conclusions on BEV PRRs

Bacterial extracellular vesicles are potent activators of PRRs in charge of regulating inflammatory responses that are correlated with pathogenesis in systemic diseases (Tiku and Tan, 2021). Additionally, pathogens release vesicles during infections to deliver virulence factors and evade immune defenses, whereas EVs from probiotics may exert a protective effect on LPS-mediated inflammation in the host (Hu et al., 2021). Thus, the balance between proinflammatory and anti-inflammatory signaling generated by PRRs upon BEV activation is crucial to understanding host–microbe interactions. Moreover, the functions of PRRs in complex disease conditions deserve in-depth studies to facilitate the design of PRR antagonists to restrict BEV-mediated inflammation in systemic diseases.

## 4. Physiological and pathological roles of BEVs in specific systems and diseases

In contrast to their parent bacteria, BEVs carry a higher concentration of virulence factors and insulate them during delivery to different organs and vascular-based tissue targets. These properties allow BEVs to travel long distances and access tissues that their parent bacteria cannot reach, strengthening the pathogenic functions of bacteria in both the local microenvironment and distant parts of the body and leading to the occurrence of Alzheimer’s disease (AD), metabolic diseases, cardiovascular disease (CVD), osteoporosis, etc.

### 4.1. Nervous system

#### 4.1.1. BEV-related neurologic disorders

**Figure 3:**
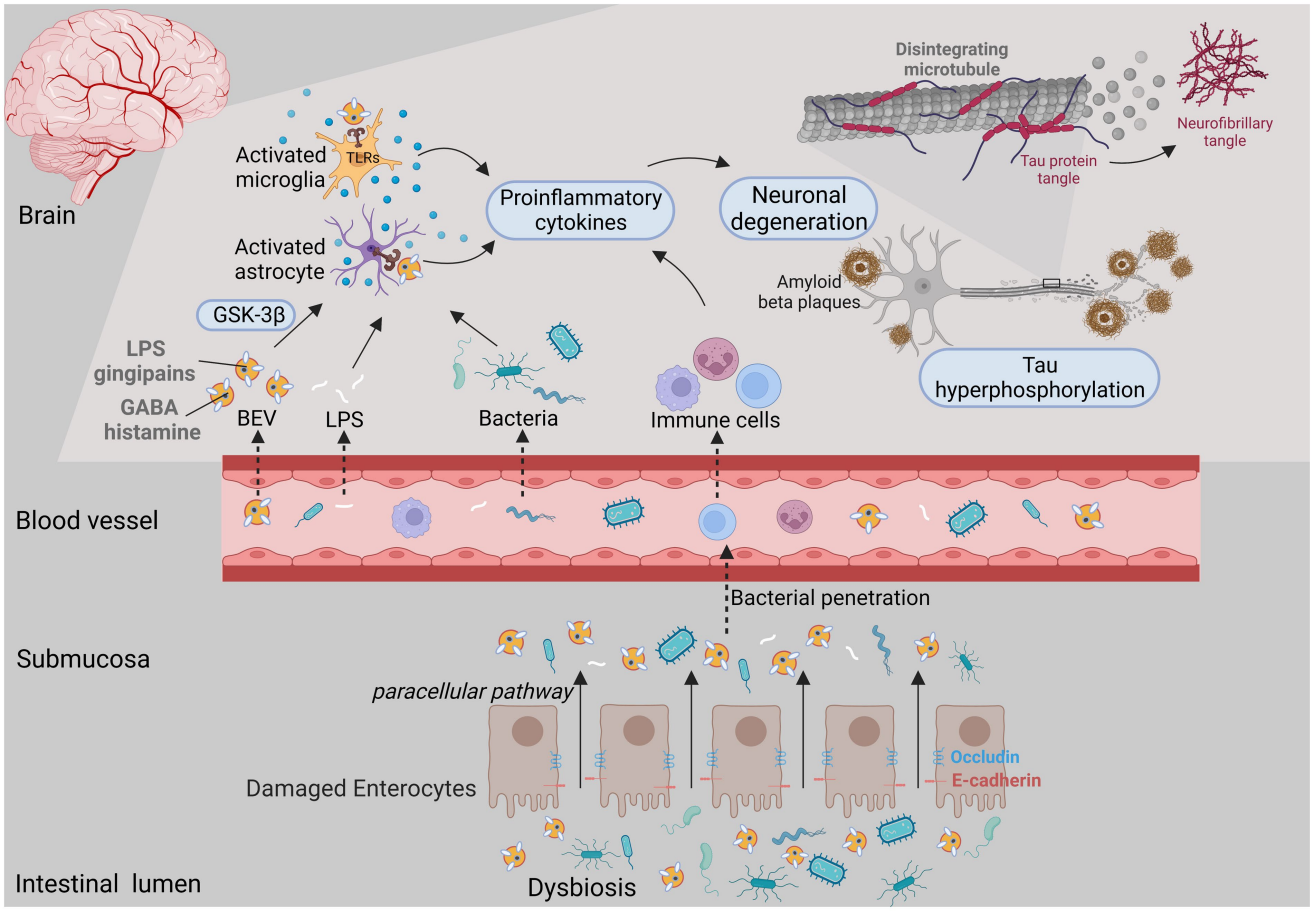
BEVs released under microecology dysbiosis could result in brain disorders. The intestinal epithelium is destroyed by both bacterial activity and the host immune response, which facilitates the penetrability and delivery of BEV cargo (e.g., LPS, gingipains, histamine and peptidoglycan) from the gut lumen to the circulation. When BEVs access the central nervous system, they potentially activate immune cells such as astrocytes and microglia through PRRs, thereby promoting proinflammatory cytokine secretion and neuronal damage and leading to neurological disorders.

Recent research have revealed the role of microbiome on neuropsychiatric disorders (Jiang et al., 2015). Individuals suffering from stress response and depressive disorder tend to have lower abundance of beneficial intestinal bacteria with their functional impairment (Aizawa et al., 2016). EVs derived from microbiome cargo a range of bioactive compounds from bacteria to affect the central nervous system function. BEVs can enter the bloodstream and permeate the blood–brain barrier (BBB) to reach the brain, subsequently affecting the regulation of various signal transduction pathways and resulting in neurologic abnormalities (e.g., dementia, AD) (Han et al., 2019; Bittel et al., 2021; Xie et al., 2023; Figure 3). BEVs can compromise the integrity of tight junctions, the disruption of which facilitates the paracellular and/or transcellular pathways of endothelial cells and promotes the delivery of BEV contents to the circulation, as well as the vagus nerve (Stentz et al., 2018; Lee et al., 2020). *Campylobacter jejuni* OMVs have been reported to cleave occludin and E-cadherin, promoting intestinal penetrability and paracellular pathways (Elmi et al., 2016). Likewise, periodontal pathogen-derived EVs enriched in gingipains, LPS and small extracellular RNAs (exRNAs) can disrupt the tight junction zona occludens protein (ZO-1) in human brain microvascular endothelial cells and cross the BBB (Han et al., 2019; Pritchard et al., 2022).

When BEVs access the central nervous system, they not only affect the reactivity of glial cells to facilitate neuroinflammation, but also exacerbate neuronal dysfunction and tau hyperphosphorylation, accelerating cognitive decline (Cheon and Lee, 2021; Park and Tsunoda, 2022). Gingipain-positive *P. gingivalis* OMVs can reach the cerebral ventricle, promote intracerebral inflammation *via* complement activation, trigger the NLRP3 inflammasome, and increase the expression of amyloid beta (Aβ) and neurofibrillary tangles (Han et al., 2019; Gong et al., 2022; Yoshida et al., 2022). Likewise, *H. pylori* OMVs led to the activation and migration of microglia and astrocytes, which induced neuronal damage *via* complement component 3 (C3)-C3a receptor (C3aR) signaling, ultimately leading to aggravated Aβ pathology (Park and Tsunoda, 2022; Xie et al., 2023). *In vitro* experiments have shown that exRNAs delivered *via* BEVs increased the expression of IL-6 in brain monocytes/microglia by activating the NF-κB pathway (Han et al., 2019; Ha et al., 2020). BEVs are also able to transport their cargo such as neurotransmitters [e.g., histamine and gamma-amino-butyric acid (GABA)], from the gut to the brain, suggesting their potential effects on brain functions (Zakharzhevskaya et al., 2017; Bittel et al., 2021). Furthermore, oral gavage of EVs derived from *Paenalcaligenes hominis* reduced brain-derived neurotrophic factor (BDNF) expression in hippocampal neurons while increasing IL-1β expression in the blood (Lee et al., 2020).

Therefore, it could be speculated that the export of BEVs to the brain may contribute to infection at any place in the body, which could result in immune responses in the brain and related neurological disorders.

#### 4.1.2. BEVs and psychiatric disease therapy

Emerging evidence confirms that pathogenic BEVs exert harmful effects on the brain function, whereas probiotic BEVs show beneficial effects on peripheral tissues. After the induction of depression symptoms by glucocorticoid (GC) treatment, *Lactobacillus plantarum*-derived EVs enhanced the expression of BDNF in the hippocampus and exerted antidepressive-like effects (Choi et al., 2019). In chronic restraint stress (CRS)-treated mice, parenterally injected EVs from *L. plantarum*, *Bacillus subtilis*, and *A. muciniphila* exhibited antidepressant-like effects and reversed stress-induced decreases in the expression of *Bdnf*, *Nt3*, and/or *Nt4/5* in HT22 cells and in the hippocampus (Choi et al., 2022). Moreover, *A. muciniphila* EVs increased 5-HT synthesis by increasing *Tph2* expression in the brain and produced a stronger effect than the parent bacterium on the reuptake and clearance of serotonin (Yaghoubfar et al., 2020). These results indicate that EVs derived from probiotics may act on neuronal cells, promote the transcription of neurotrophic factors, and produce antidepressant-like effects, which have the potential to be applied to the design of neuropsychiatric treatment such as drug delivery vehicles and vaccines.

### 4.2. Digestive system

#### 4.2.1. Oral cavity

##### 4.2.1.1. *Streptococcus mutans* MVs and caries

Since *S. mutans* MVs can disseminate over long distances, their local exploitation of nutrient substances such as sucrose to produce extracellular polysaccharides (EPS) on the hydroxyapatite surface could facilitate bacterial colonization and biofilm formation associated with cariogenicity (Nakamura et al., 2020). *S. mutans* MVs have been found to package metabolic enzymes associated with carbohydrate metabolism, such as glucosyltransferase (Gtf), glucan-binding proteins and dextranase (DexA) (Cao et al., 2020). Moreover, *S. mutans* MVs containing Gtfs increase EPS formation in *C. albicans* biofilms, and genes of *C. albicans* related to mannan and glucan synthesis increased upon exposure to *S. mutans* MVs, indicating that *S. mutans* MVs facilitate cariogenic bacterial carbohydrate metabolism (Wu et al., 2020).

MVs also promote the cariogenic ability of bacteria even at low pH values. Intriguingly, the initial pH value affects various characteristics of *S. mutans* MVs, including biofilm quantity (Nakamura et al., 2020; Iwabuchi et al., 2021). Under low pH conditions, *S. mutans* released more MVs to deliver proteins related to cariogenesis, and several important enzymes carried by MVs, such as the shock heat proteins, lactate dehydrogenase, DexA and Gtfs, still possessed enzyme activity (Cao et al., 2020). These may be new mechanisms of MV biogenesis and could underlie the acid resistance of *S. mutans*; furthermore, these data are helpful to develop biofilm formation inhibitors targeting BEVs to prevent dental caries.

##### 4.2.1.2. Periodontitis

Once released, periodontopathogen-derived EVs, enriched in virulence factors such as muramic acid, LPS, fimbriae, dentilisin, outer membrane proteins and gingipains, may act as representatives of parent bacteria to communicate with other oral bacteria and host cells and adhere to the tooth surface (Inagaki et al., 2006). For instance, *P. gingivalis* EVs alone can promote the aggregation of a broad range of *Streptococcus* spp.*, Fusobacterium nucleatum, Treponema denticola, Actinomyces viscosus, Actinomyces naeslundii,* and *Lachnoanaerobaculum saburreum* in oral biofilms (Hiratsuka et al., 1992; Kamaguchi et al., 2003; Inagaki et al., 2006; Grenier, 2013). *P. gingivalis* EVs aggregate other oral bacteria primarily depending on OMV-related gingipain proteases (Ito et al., 2010). Additionally, other species present in oral biofilms, such as *T. forsythia*, can also release OMVs related to biofilm formation (Friedrich et al., 2015). BEVs also protect other organisms from complement activities to accelerate the progression of periodontitis. Consistent with this, *Actinobacillus actinomycetes* EVs can serve as decoys to activate complement in an LPS-dependent manner and deplete complement to defend against serum-susceptible bacteria (Lindholm et al., 2020). Moreover, *P. gingivalis* OMVs induced selective TNF deficiency that suppressed microbial recognition by macrophages/monocytes (Waller et al., 2016). Apart from escaping the surveillance of innate immune cells, BEVs also evade adaptive immune cells. For instance, small RNAs carried by OMVs derived from *A. actinomycetemcomitans*, *P. gingivalis*, and *T. denticola* inhibited the release of IL-13 and IL-5 by Jurkat T cells (Choi et al., 2017). This evidence indicates the contribution of periodontal pathogenic EVs to bacterial survival and aggregation, favoring the pathogenic process of periodontitis.

Bacterial extracellular vesicles can activate the first guard against bacterial infections, the oral mucosal epithelium, in multiple ways. *P. gingivalis* EVs can be internalized into epithelial and endothelial cells *via* lipid raft-mediated endocytosis and facilitate the invasion of other pathogens, such as *Tannerella forsythia* (Furuta et al., 2009). After invasion, BEVs inhibit oral epithelial migration and proliferation, leading to cell dysfunction in periodontal tissues (Furuta et al., 2009). For example, *P. gingivalis* OMVs lead to apoptosis after their uptake by human periodontal ligament cells and cause pyroptosis by activating inflammasomes both *in vitro* and *in vivo* (Cecil et al., 2017; Fan et al., 2023).

After their evasion of the oral epithelial barrier, BEVs enter submucosal tissues, where they interact directly with host innate or adaptive immune cells. OMVs released from *P. gingivalis*, *T. forsythia* and *T. denticola*, activate PRRs on macrophages and monocytes, and increase the production of TNF-α, IL-1β, and IL-8 (Cecil et al., 2016). Similarly, OMVs from *A. actinomycetemcomitans* activated NOD1-dependent nuclear factor kappa-B (NF-κB) in monocytes (Thay et al., 2014). OMVs from *F. nucleatum* also facilitated the differentiation of macrophages toward the proinflammatory phenotype (Chen et al., 2022). Moreover, OMVs may be a second route through which neutrophils in the oral cavity may encounter bacterial virulence factors and hinder neutrophil chemotaxis and phagocytosis (Jones et al., 2019). It is possible that the inflammatory milieu induced by BEVs can further exacerbate their toxicity to gingival fibroblasts and periodontal tissue destruction.

Bacterial extracellular vesicles can deliver toxic payloads to susceptible cells in the periodontium and aggravate alveolar bone loss, thereby causing periodontal tissue destruction. *A. actinomycetemcomitans* OMVs were found to promote damage in the sulcular/junctional epithelium *via* the delivery of cytolethal distending toxin into human gingival fibroblasts (Rompikuntal et al., 2012). A recent study reported that EVs from both oral commensal bacteria and periodontal pathogens can provoke osteoclastogenic activity through TLR2 activation (Kim et al., 2022). Intracellular delivery of prostaglandin (PG) *via A. actinomycetemcomitans* OMVs could directly trigger alveolar bone loss (Jiao et al., 2013). Likewise, *F. nucleatum* BEVs increased osteoclast numbers, and inflammatory factor (IL-1β, IL-6, and TNF-α) production, and accelerated periodontal bone loss in a periodontitis mouse model (Chen et al., 2022).

In conclusion, periodontopathogen-derived vesicles can activate or degrade bioactive substances in host cells, hinder cell proliferation, facilitate cell death, and induce inflammatory cytokine release, thereby promoting the establishment of an inflammatory microenvironment in periodontal tissues and subsequent alveolar bone destruction.

#### 4.2.2. Liver

##### 4.2.2.1. BEVs and diabetes mellitus

**Figure 4:**
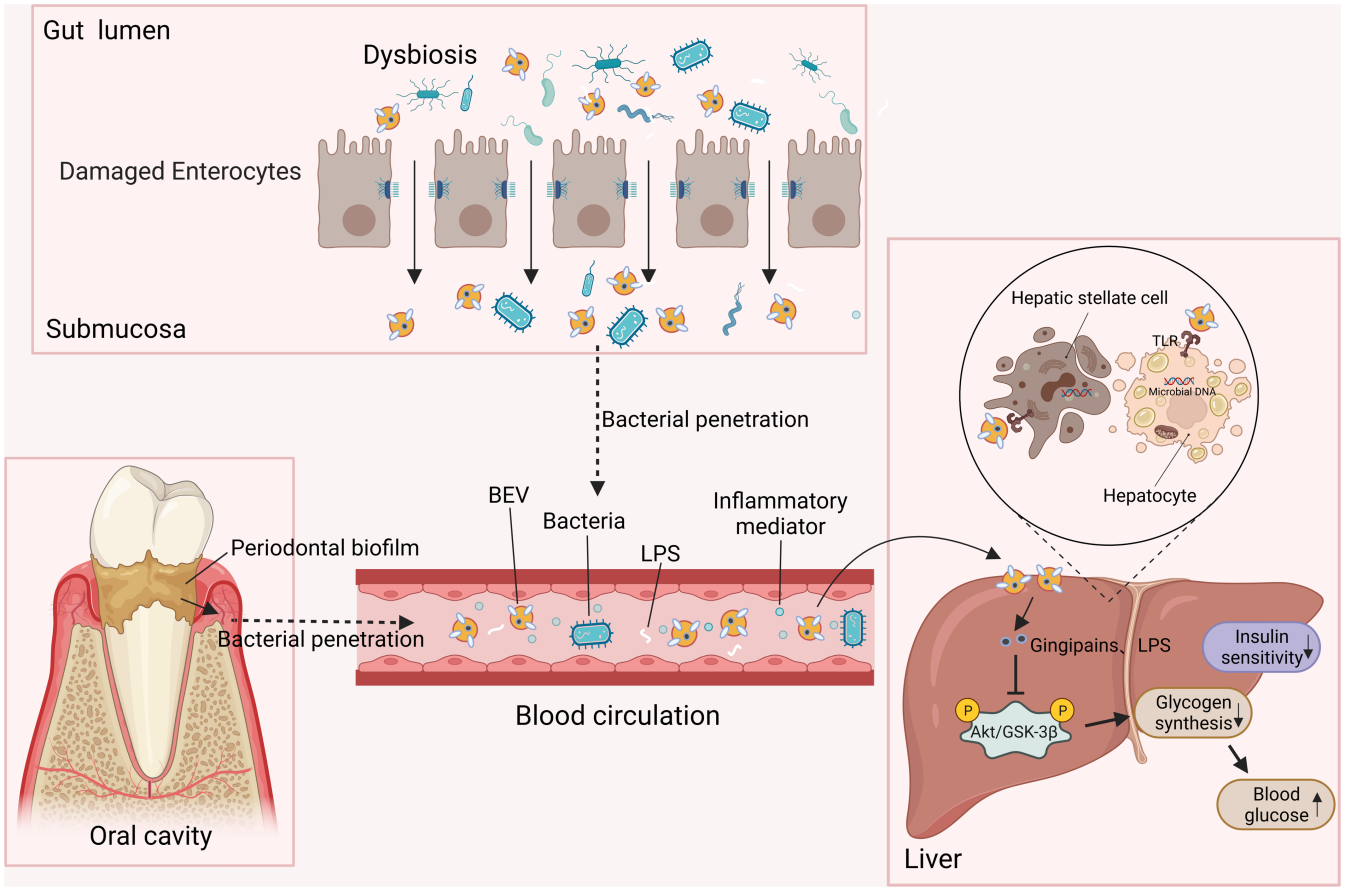
BEVs participate in insulin resistance and result in T2DM. EVs from periodontal biofilms and dysfunctional intestinal microbiota deliver toxins such as gingipains and LPS to the liver through the circulation. When they access the liver, BEVs induce insulin resistance in hepatic stellate cells and hepatocytes by inhibiting AKT/GSK-3β signaling, decreasing hepatic glycogen synthesis, and ultimately increasing the level of blood glucose.

Bacterial extracellular vesicles have been recently considered a critical mediator facilitating the pathogenic process of the endocrine system disease type 2 diabetes mellitus (T2DM), and they can also be applied to the diagnosis and treatment of T2DM and its complications (Figure 4). A significantly higher concentration of BEVs was observed in patients with T2DM than in the healthy population among stool, serum, and urine (Nah et al., 2019). In diabetes animal model, intestinal microbiota-derived OMVs are also increased (Chen et al., 2023). Furthermore, gut microbe-derived EVs were reported to permeate the intestinal barrier and enter the bloodstream followed by distribution to distant metabolic organs (e.g., adipose tissue, liver, and skeletal muscle), where they trigger insulin resistance and damage glucose metabolism (Choi et al., 2015; Nah et al., 2019; Bittel et al., 2021). For instance, *P. panacis* OMVs can block insulin signaling in adipose and skeletal tissue, and induce a diabetic phenotype in mice (Choi et al., 2015). Gingipain-positive cells were found in the liver sinuses of mice injected with *P. gingivalis* OMVs, suggesting that the hepatic cells were exposed to gingipains delivered by OMVs (Seyama et al., 2020). In these mice, gingipains in *P. gingivalis* OMVs weakened glycogen synthesis and insulin sensitivity through the activated protein kinase B (Akt)/glycogen synthase kinase-3 beta (GSK-3β) signaling pathways (Nakayama et al., 2015; Seyama et al., 2020). Moreover, obese BEVs enriched in microbial DNA notably lowered the number of liver CRIg+ and islet Vsig4+ macrophages, causing the dissemination of BEVs to insulin-responsive tissues and subsequently aggravating the inflammation and insulin resistance of hepatocytes through the activation of cGAS/STING signaling (Luo et al., 2021; Gao et al., 2022). These studies emphasized that BEVs either from the oral or intestinal microbiota, as participants in insulin resistance, are correlated with obesity and an increased incidence of T2DM.

##### 4.2.2.2. Probiotic-derived EVs improve metabolic function

Detrimental BEV characteristics are counterbalanced with their beneficial characteristics under physiological and pathological circumstances given that probiotic-derived EVs prevent adverse processes that induce obesity-related diseases. For instance, *Akkermansia muciniphila*-derived EVs enhanced the expression of tight junction proteins in Caco-2 cells and eventually increased gut barrier integrity in an HFD-induced diabetic model through AMPK activation (Ashrafian et al., 2021). Treatment with *A. muciniphila* OMVs markedly ameliorated lipid metabolism and reduced inflammatory cytokine release in adipose tissues (Ashrafian et al., 2019). *A. muciniphila* and its OMVs could also regulate energy balance and improve blood parameters, such as lipid profiles and glucose levels (Ashrafian et al., 2019). The aforementioned data indicate that probiotic-derived EVs can potentially be used to improve intestinal penetrability and metabolic functions such as glucose and lipid metabolism, while more investigation related to the underlying mechanism is needed to treat obesity-related diseases.

##### 4.2.2.3. BEVs and nonalcoholic fatty liver disease

BEVs may be involved in several mechanisms, such as intestinal barrier disruption and systemic inflammation, associated with the onset and progression toward nonalcoholic fatty liver disease (NAFLD) or nonalcoholic steatohepatitis (NASH)-related liver abnormalities. Intragastrically administered feces-derived EVs (fEVs) entered the liver and increased proinflammatory cytokines and chemokines from hepatic sinusoidal endothelial cells *via* TLR4 action through LPS and activated profibrotic and proinflammatory protein production in hepatic stellate cells (Fizanne et al., 2023). Likewise, LPS-positive *P. gingivalis* EVs provoked Kupffer cell (KC) activation through TLR4 and subsequent liver inflammation, glycogen synthesis reduction and progression toward steatohepatitis (Miura et al., 2010). *H. pylori* OMVs increased the level of liver fibrosis markers in hepatocytes, and exosomes derived from OMV-treated cells activated hepatic stellate cells (HSCs) and induced liver fibrosis (Zahmatkesh et al., 2022). Furthermore, the accumulation of microbial DNA may be a mechanism involved in NAFLD progression. Intestinal BEV translocation promoted bacterial DNA accumulation in HSCs and hepatocytes, which induced hepatocyte inflammation and HSC fibrosis *via* the activation of cGAS/STING (Luo et al., 2022). Collectively, BEVs may be a critical moiety in the pathogenesis of NAFLD by facilitating liver inflammation and hepatic steatosis and fibrosis by delivering toxic payloads into liver cells.

##### 4.2.2.4. The antifibrotic effects of BEVs

Probiotic-derived vesicles exhibit beneficial effects on the prevention of liver inflammation and liver fibrosis. Research has shown that *A. muciniphila* EVs could efficiently enhance the regression of activated HSCs (Keshavarz Azizi Raftar et al., 2021). In the HFD/carbon tetrachloride-induced liver injury model, *A. muciniphila* OMV treatment substantially attenuated fibrosis and inflammatory biomarkers and ameliorated liver and colon damage (Keshavarz Azizi Raftar et al., 2021). Therefore, EVs from probiotics may have anti-inflammatory and antifibrotic effects and protect against liver injury.

#### 4.2.3. Gut

##### 4.2.3.1. BEVs and inflammatory bowel disease

Bacterial extracellular vesicles exhibit regulatory effects on intestinal immunity and homeostasis, as evidenced by stool BEVs from an IBD mouse model showing severe dysbiosis compared to that of the normal controls (Kang et al., 2013). BEVs were also implicated in barrier damage in IBD, HIV and cancer therapy-induced intestinal mucositis, leading to an intestinal and systemic inflammatory environment in these diseases (Liu et al., 2021).

Bacterial extracellular vesicles released into the intestinal lumen can pass through the mucus layer and interact with intestinal epithelial cells and immune cells, regulating immunomodulation and corresponding signaling pathways in the pathogenesis of IBD (Gul et al., 2022). In colonic epithelial cells and human colonoid (organoid) monolayers, *F. nucleatum*-derived OMVs activate TLR4, leading to inflammatory cytokine release (Engevik et al., 2021). Studies that focused on the effects of BEVs on the mucosal immune system showed that the uptake of *B. fragilis* OMVs by dendritic cells (DCs) induced regulatory T cells (Tregs), and *Bacteroides thetaiotaomicron* OMVs also exerted an effect on T-cell functions (Chu et al., 2016; Wegorzewska et al., 2019). Likewise, OMVs from specific strains of *E. coli* activated DCs and derived CD4+ T-cell responses (Diaz-Garrido et al., 2022). In patients with UC and CD, a lack of regulatory IL-10 response by DCs to *B. thetaiotaomicron* OMVs was observed (Durant et al., 2020). Efficient BEV internalization by mucosal phagocytic cells both *in vitro* and *in vivo* occurred and pronounced BEV-induced inflammatory responses in these macrophages were observed (Bittel et al., 2021). The regulation of immunomodulatory miRNAs by BEVs may partly underlie several specific effects (Díaz-Garrido et al., 2020). For instance, *B. thetaiotaomicron* EVs have been found to harbor microbial helicases specifically targeting the human polymerase protein PAPD5, a negative regulator of miR-21, the targets of which are genes that participate in immune responses and the pathogenesis of IBD (Gul et al., 2022).

In general, these studies illustrate that gut microbe-derived EVs distributed in serum or other tissues could be an effective marker for intestinal integrity and could promote inflammation in the gut and even in distant organs through the leaky intestinal barrier (Nah et al., 2019). Further research is needed to elucidate the systemic functions of circulating BEVs and to determine and associate their taxonomy with the metabolic activity of the gut microbiota.

##### 4.2.3.2. BEVs and intestinal viral infection

The significance of BEVs is further consolidated by the capability of enteric viruses to utilize these vesicles to facilitate viral infection. EVs from commensal *Enterobacter cloacae*, *B. thetaiotaomicron*, and *Lactobacillus acidophilus* cross the intestinal epithelium and enter the lamina propria in which the prime targets of acute norovirus infection, namely, immune cells, reside (Mosby et al., 2022, 2023). Similarly, *P. gingivalis* OMVs promoted HIV translocation from mucosal surfaces to subcutaneous tissues and reached HIV permissive cells, such as DCs and T cells, and nonpermissive cells, such as human oral keratinocytes; this may also serve as a mechanism for cell-free HIV transcytosis through the intestine (Dong et al., 2018). Moreover, virus interaction with commensal bacteria changes the size, yield, cargo and content of BEVs, suggesting that viral binding may alter the mechanism of BEV biogenesis (Mosby et al., 2022, 2023). Therefore, BEVs potentially offer a mechanistic basis for the bacterial promotion of viral infection by facilitating virus entry into target cells and regulating host immune responses; meanwhile, the relevant mechanism deserves further exploration for a better understanding of bacteria-virus infections and to develop beneficial therapeutic strategies.

##### 4.2.3.3. BEVs and cancer development

Several studies have demonstrated that BEVs can penetrate the intestinal epithelial barrier, selectively accumulate near intestinal tumor cells, change the tumor microenvironment (TME), and participate in the progression of gastrointestinal cancer. *H. pylori*-derived OMVs were upregulated in the gastric juice of gastric cancer patients compared to healthy controls, and could penetrate and remain in the mouse stomach for an extended period of time (Choi et al., 2017). Intravenous injection of *E. coli* OMVs specifically accumulated near the tumor tissues of BALB/c mice with CT26 tumors (Kim et al., 2017). These vesicles attract T cells and natural killer cells, and induce the production of TNF-α, IL-6, and IL-1β by macrophages and IL-8 by gastric epithelial cells (Choi et al., 2017; Kim et al., 2017). EVs from *E. coli* could be internalized into the Caco-2 cell line and promote carcinogenesis in intestinal epithelial cells (Tyrer et al., 2014). Likewise, OMVs from *E. coli* and *Vibrio cholerae* were involved in enhancing cell differentiation in colon cancer cells (Vdovikova et al., 2018). *H. pylori* OMVs were found to contain CagA and VacA proteins, which were correlated with the induction of apoptosis in the adenocarcinoma gastric cell line (AGS) and an increase in ATP affinity to H1 histone proteins in chromosomes (Turkina et al., 2015). Moreover, BEVs can increase the release of proinflammatory cytokines and activate a series of abnormal signaling pathways, leading to the occurrence of cancer (Choi et al., 2017). Therefore, BEVs can not only access the TME efficiently but also alter the TME by producing or inducing the release of oncogenic metabolites. Furthermore, the composition of intestinal microbe-derived EVs in colorectal cancer exhibited discrepancies compared to that of healthy controls, indicating that BEVs may be harnessed as a marker for detecting cancer and predicting cancer prognosis (Park et al., 2021).

##### 4.2.3.4. BEVs help maintain intestinal homeostasis

Bacterial extracellular vesicles released by probiotic and commensal bacteria have been indicated to activate the immune system and maintain gut homeostasis in multiple ways (Table 2). BEVs can regulate the interaction with host cells by regulating PRRs. For instance, OMVs derived from *B. fragilis* modified the gene expression of TLR2 and TLR4 in epithelial cells and increased the secretion of IL-10 by CD4^+^ T cells (Ahmadi Badi et al., 2019). DCs sense OMV-associated polysaccharides through TLR2, resulting in an increase in Tregs and anti-inflammatory cytokine production (Shen et al., 2012). Furthermore, epithelial cells could sense OMVs derived from commensal *E. coli* strains ECOR12 and Nissle 1917 in a NOD1-dependent manner and regulate cytokine production (Cañas et al., 2018).

**Table 2:** Contribution of gut microbe EVs to immune homeostasis.

Species	Evidence from studies	Reference
*Escherichia coli* Nissle 1917	Reduction in the expression of pro-inflammatory cytokines in colitis	Lee et al. (2012)
	Increase in epithelial barrier integrity through the upregulation of tight junction proteins	Alvarez et al. (2016)
*Bacteroides fragilis*	Promotion of an immunomodulatory Treg response through DCs stimulation in colitis and mucosal tolerance through the regulation of autophagic genes	Shen et al. (2012)
	Induction and inhibition of anti-inflammatory and pro-inflammatory cytokines in the Caco-2 cell line, respectively	Ahmadi Badi et al. (2019)
*Lactobacillus rhamnosus*	Increase in gut DC levels and the induction of IL-10 release	Al-Nedawi et al. (2015)
*Lactobacillus sakei*	Increase in IgA production in the gut and improvement in epithelial barrier function	Yamasaki-Yashiki et al. (2019)
*Lactobacillus kefir, Lactobacillus kefiranofaceins, Lactobacillus kefirgranum*	Suppression of proinflammatory cytokine production in an IBD mouse model	Seo et al. (2018)
*Akkermansia muciniphila*	Inhibition of colitis progression by improving macroscopic scores	Kang et al. (2013)
	Promotion of AMPK phosphorylation and prevention of LPS-induced intestinal barrier damage	Chelakkot et al. (2018)
	Recovery of the gut barrier integrity in HFD-induced obesity by improving the expression of tight junction proteins	Chelakkot et al. (2018)
*Bifidobacterium longum*	Improvements in allergic diarrhea through mast cells apoptosis in a food allergy mouse model	Kim et al. (2016)
*Bifidobacterium bifidum*	Promotion of an immunomodulatory Treg response through DC stimulation in PBMCs-isolated naïve T cells	López et al. (2012)
*Bifidobacterium vulgatus*	Induction of tolerance in colonic BMDCs	Maerz et al. (2018)

DCs, dendritic cells; IL, interleukin; HFD, high-fat diet; IBD, inflammatory bowel disease; IgA, immunoglobulin A; PBMCs, peripheral blood mononuclear cells; BMDCs, Bone Marrow-Derived Dendritic Cells.

In mouse models, EVs from *Bifidobacterium longum* and *Bifidobacterium bifidum* dampened allergy-related diarrhea by inducing mast cell apoptosis and Treg production, respectively (López et al., 2012; Kim et al., 2016). Additionally, oral treatment with MVs from *Lactobacillus rhamnosus* promoted the expression of IL-10 and heme oxygenase-1 in bone marrow-derived DCs and then triggered Tregs in Peyer’s patches and mouse mesenteric lymph nodes (Al-Nedawi et al., 2015). Likewise, *B. thetaiotaomicron* OMVs mediated monocyte activation and IL-10 production through TLR2 activation and alleviated acute intestinal inflammation in dextran sodium sulfate (DSS)-treated mice (Fonseca et al., 2022). *Bacteroides vulgatus* and *B. fragilis* OMVs have also been reported to elicit a tolerogenic phenotype in DCs and enhance Treg production, respectively (Shen et al., 2012; Maerz et al., 2018). These studies indicate the potential utilization of BEVs to reinduce tolerance and rebuild immune homeostasis in IBD.

Regarding the protective effects on restoring the integrity of the physicochemical barrier, OMVs released by *E. coli* Nissle 1917 could reduce inflammation in DSS-treated mice and increase IL-22 in colonic explants (Alvarez et al., 2016; Fábrega et al., 2017). *A. muciniphila* OMVs also decreased inflammatory cell recruitment to the colon wall in DSS-induced colitis and restored epithelial stability by promoting the expression of tight junctions and mucus in epithelial cells (Kang et al., 2013; Wang et al., 2023). In 2,4,6-trinitrobenzenesulfonic acid (TNBS)-induced IBD, MVs from several *Lactobacillus* species, namely, *kefir*, *kefirgranum*, and *kefiranofaceins*, were demonstrated to reduce the release of proinflammatory cytokines (Seo et al., 2018). *A. muciniphila* OMVs also suppressed HFD-induced colonic inflammation, increased AMPK phosphorylation and prevented LPS-induced intestinal barrier damage (Chelakkot et al., 2018; Ashrafian et al., 2021). Moreover, MVs from *Lactobacillus sakei* and *A. muciniphila* promoted the production of IgA in the intestine and improved epithelial barrier function (Yamasaki-Yashiki et al., 2019; Wang et al., 2023). Furthermore, BEV-mediated modulation of the intestinal microbiota might involve selective cross-talk with specific commensal species, as indicated by *A. muciniphila* OMV-mediated increase in the abundance of beneficial commensal Firmicutes and Bacteroidetes and decrease in the abundance of potentially pathogenic taxa in the phylum Proteobacteria (Wang et al., 2023).

Collectively, the multifunctional role of BEVs in modulating intestinal homeostasis may occur through reciprocal and complementary mechanisms that regulate mucosal immunity, physicochemical barriers, and the gut microbiota. Thus, enteric microbiota-derived EVs may provide insight into therapeutic strategies against diseases implicated in inflammation and barrier dysfunction, such as T2DM.

### 4.3. Circulatory system

Bacterial extracellular vesicle is a hazard factor for CVD and coronary heart diseases such as atherosclerosis, among which endothelial dysfunction and calcium deposition play a key role in the development of atherosclerosis. Nanoscale BEVs can lead to proteolytic damage in blood vessels that cannot be accessed by bacteria, making them analogous to parent bacteria in the pathogenesis of CVD (Farrugia et al., 2020).

Studies have shown the role that BEVs play in causing endothelial injury to promote vascular permeability and cause disease phenotypes both *in vitro* and *in vivo* (Zhang et al., 2020). *P. gingivalis* OMVs can increase vascular permeability probably through proteolytic cleavage of endothelial cell–cell adhesins such as PECAM-1 (Farrugia et al., 2020). *P. gingivalis* OMVs can also activate Rho kinase (ROCK) in human umbilical vein endothelial cells, causing endothelial dysfunction (Jia et al., 2015). In addition, stimulation with OMVs from CagA-enriched *H. pylori* facilitated atherosclerotic plaque formation *via* endothelium injury *in vivo* and promoted apoptosis in human umbilical vein endothelial cells (Wang et al., 2021). Additionally, *P. gingivalis* OMVs induced the calcification of vascular smooth muscle cells by activating the ERK1/2-RUNX2 pathway (Miyakawa et al., 2004; Yang et al., 2016).

Bacterial extracellular vesicles facilitate cardiac tissue inflammation to cause related diseases. A recent study revealed that gut BEVs containing microbial DNA led to obesity-associated adrenomedullary inflammation and catecholamine production (Gao et al., 2022). Additionally, EVs from a uropathogenic *E. coli* strain exerted a direct effect on cardiomyocytes and induced cardiac tissue inflammation and injury (Svennerholm et al., 2017).

These studies highlight the idea that the entry of BEVs into circulation potentially initiates atherosclerosis and cardiac tissue inflammation and may contribute to the disruption of the vascular system, resulting in the occurrence of CVD.

### 4.4. Respiratory system

The existence of BEVs in the lungs of patients with severe pulmonary infections and the fact that BEVs can transport virulence factors may indicate their role in the process of infection (Bomberger et al., 2009). The mechanism mediated by BEVs may inhibit the host’s immune response to bacteria. For example, the delivery of Cif through *P. aeruginosa* OMVs to the cytoplasm of host cells hampered CTRF chloride production and thus dampened the ability to clear respiratory pathogens through mucus cilia (Bomberger et al., 2011). *P. aeruginosa* OMVs also evade the host immune response by altering DNA methylation in human lung macrophages (Kyung Lee et al., 2021). *Streptococcus pneumoniae* MVs delivered vesicle-associated proteins into human monocyte-derived dendritic cells, induced proinflammatory cytokines, and exposed targets for complement factors in serum, thereby promoting pneumococcal evasion of humoral host defense (Codemo et al., 2018).

It has been recently shown that the majority of host proinflammatory responses induced by PAMPs are mediated by BEVs. MVs produced by *S. aureus* also fuse in a cholesterol-dependent manner with the plasma membrane of host cells, causing the delivery of α-hemolysin (HIa), which can trigger apoptosis in T-lymphocytes (Thay et al., 2013). *Klebsiella pneumoniae* OMVs increased the proinflammatory cytokines IL-1β, IL-8 and TNF-α in human epithelial cells, mast cells and macrophages (You et al., 2019). Likewise, OMVs secreted by respiratory pathogens induced a strong proinflammatory response in immature THP-1 macrophages (Volgers et al., 2017). *L. pneumophila* OMVs can activate macrophages *via* TLR2 and cause tissue damage in human lung tissue explants (Jäger et al., 2015; Jung et al., 2016). Furthermore, peptidoglycan-containing OMVs were internalized into epithelial cells *via* lipid rafts to trigger NOD1-dependent responses both *in vitro* and *in vivo* (Kaparakis et al., 2010). Additionally, intratracheal exposure to *K. pneumoniae* OMVs caused severe lung pathology in neutropenic mice similar to bacterial infection (Lee et al., 2012). OMVs from *P. aeruginosa* and *A. baumannii* provoked pulmonary inflammation *in vivo*, partly modulated by the TLR2 and TLR4 pathways (Park et al., 2013; Marion et al., 2019). Furthermore, BEVs can promote the development of airway hypersensitivity to inhaled allergens. Repeated airway treatment with *S. aureus* MVs provoked Th1 and Th17 neutrophilic pulmonary inflammation, primarily through TLR2 signaling (Kim et al., 2012).

In addition, BEVs can promote bacterial colonization in the respiratory tract and the maintenance of biofilms. For instance, EVs from several common respiratory pathogens including, *Haemophilus influenzae, M. catarrhalis, S. pneumoniae, and P. aeruginosa,* promoted the adherence and aggregation of intracellular bacteria (Volgers et al., 2017). The changes induced by *P. aeruginosa* OMVs resulted in an increase in the Psl/biomass ratio in the early biofilm matrix, which helped to protect growing colonies from the harmful effects of antimicrobial agents (Esoda and Kuehn, 2019). Additionally, *C. albicans* biofilm EVs participated in matrix polysaccharide formation and decreased sensitivity to the antifungal drug fluconazole (Zarnowski et al., 2018). Furthermore, a proteomics study of *P. aeruginosa* biofilms identified that the proteins related to OMVs consist of more than 20% of the total matrix proteome (Couto et al., 2015). Many proteins associated with virulence are exclusively secreted *via L. pneumophila* OMVs, such as intracellular survival and replication (ProA1), invasion (IcmK), persistence and spreading in the lung (fliC) (Galka et al., 2008). Furthermore, after exposure to OMVs, vitronectin increased both *in vivo* and *in vitro*, and the increase in vitronectin in the bronchoalveolar space helped evade complement-mediated clearance (Paulsson et al., 2018).

In summary, by targeting the BEV-related contents involved in the interaction between these vesicles and human lung cells or immune cells, new treatments for pulmonary infections may emerge, such as vaccines or drugs, that protect patients from bacterial invasion.

### 4.5. Motor system

#### 4.5.1. The osteoclastic effects of BEVs

Studies have revealed that microbes or their released vesicles can induce inflammatory responses to initiate osteoclast activity and dampen osteoblast activity, leading to bone loss. Some citrullinated proteins were confirmed in OMVs from *P. gingivalis*, which implied a correlation between BEVs and rheumatoid arthritis (RA) (Larsen et al., 2020). Peptidylarginine deiminase (PPAD), which is correlated with the occurrence of RA, was also abundantly present in secreted BEVs (Gabarrini et al., 2018a,b). Moreover, human osteoblasts and synovial cells can internalize *Kingella kingae* OMVs, and the levels of granulocyte-macrophage colony-stimulating factor (GM-CSF) and IL-6 increase in RA synovial fluid upon interaction with OMVs, promoting signal transduction in infected joints and damaging bone tissues during bacterial infection (Maldonado et al., 2011). BEVs also aggravate joint damage by promoting bacterial evasion. After exposure to *P. gingivalis* OMVs, *S. aureus* accumulated in a gingipain-and PPAD-dependent manner, which promoted the uptake of *Staphylococcus* by human neutrophils and facilitated bacterial entry into the bloodstream (du Teil Espina et al., 2022). Therefore, the role of BEVs could potentially explain why RA patients show higher levels of disease severity or complications such as osteoarticular infection.

To investigate the association between BEVs and osteoporosis, an *in vivo* model of MAMP-induced inflammatory bone loss in mice was established, and *Filifactor alocis* EVs triggered systemic bone loss and osteoclastogenesis through TLR2 activation (Song et al., 2020; Kim et al., 2021). These studies provide new insight into the effects of pathogen-derived EVs in systemic bone loss.

#### 4.5.2. The osteoprotective effects of BEVs

In contrast, probiotic-derived vesicles exhibit osteoprotective effects. After oral administration to GC-treated mice, *Lactobacillus animalis* EVs could access the femoral head and improve trabecular bone microarchitecture (Chen et al., 2022). EVs produced by *A. muciniphila* and the gut microbiota from children can access and accumulate in bone tissues to ameliorate ovariectomy-induced osteoporotic phenotypes by enhancing osteogenic activity and dampening osteoclast formation (Liu et al., 2021). Likewise, *Proteus mirabilis* OMVs inhibited osteoclast differentiation and caused mitochondria-dependent apoptosis (Wang et al., 2022). In the same study, treatment with OMVs restored bone loss in experimental osteoporosis and collagen-induced arthritis (Wang et al., 2022). Intriguingly, BEVs counteracted bacteria-mediated osteoclastogenic pathways. For example, *K. kingae* OMVs decreased osteoclastogenesis in a dose-dependent manner and inhibited proinflammatory cytokine production by infected macrophages (Pesce Viglietti et al., 2021). Therefore, BEVs exhibit advantages in bone health, and these studies offer a mechanistic basis for BEV-mediated osteoprotective functions.

## 5. Conclusion

In recent decades, our knowledge of the physiological and pathological effects of EVs derived from gram-negative and gram-positive bacteria has improved unprecedentedly. BEVs are now commonly recognized as a delivery system that consolidate bacterial roles in bacterial survival, inflammation and pathogenesis in diverse biological milieu, and bacteria can modulate the biogenesis and content of BEVs in a tailored manner as needed. Moreover, recent advances in BEV science have attempted to address the question of how BEV-host interactions contribute to systemic diseases from different perspectives. To expound the intricate mechanisms underlying the role that BEVs play in infection and anti-infection activities in almost every system, we hope to explore novel therapeutic interventions.

## Author contributions

YW: Writing – original draft. XL: Supervision, Writing – review & editing. XX: Writing – review & editing, Visualization. CH: Writing – review & editing, Validation. DM: Writing – review & editing.
